# Antimicrobial Resistance Patterns and Prevalence of *blaPER-1* and *blaVEB-1 *Genes Among ESBL-producing *Pseudomonas aeruginosa Isolates* in West of Iran

**DOI:** 10.5812/jjm.8888

**Published:** 2014-01-01

**Authors:** Mohammad Yousef Alikhani, Zahra Karimi Tabar, Fatemeh Mihani, Enayat Kalantar, Pegman Karami, Mahnaz Sadeghi, Shiva Ahdi Khosroshahi, Safar Farajnia

**Affiliations:** 1 Department of Microbiology, Faculty of Medicine, Hamadan University of Medical Sciences, Hamadan, IR Iran; 2 Department of Microbiology, School of Medicine, Alborz University of Medical Sciences, Alborz, IR Iran; 3 Tuberculosis and Lung Research Center, Tabriz University of Medical Sciences, Tabriz, IR Iran; 4 Biotechnology Research Center, Tabriz University of Medical Sciences, Tabriz, IR Iran; 5 Drug Applied Research Center, Tabriz University of Medical Sciences, Tabriz, IR Iran

**Keywords:** *Pseudomonas aeruginosa*, Antimicrobial Drug Resistance, Beta-lactamase

## Abstract

**Background:**

*Pseudomonas aeruginosa* is a leading cause of nosocomial infections worldwide. Resistance of *P. aeruginosa* strains to the broad-spectrum cephalosporins may be caused by extended-spectrum β-lactamases (ESBLs).

**Objectives:**

The aim of this study was to determine the antimicrobial resistance patterns and prevalence of PER-1 and VEB-1 type genes among ESBL producing strains of *P. aeruginosa*.

**Material and Methods:**

A total of 106 *P. aeruginosa* isolates were collected from two university hospitals in Hamadan, Iran, during a7-month study (2009). The antimicrobial susceptibility of isolates was determined by disc diffusion method and interpreted according to the clinical and laboratory standards institute (CLSI) recommendations. Production of ESBL was determined by combined disk test and presence of PER-1 and VEB-1 type ESBL genes was identified by PCR.

**Results:**

The resistance against broad-spectrum cephalosporins and monobactames were: cefepime (97%), cefotaxime (92.5%) ceftazidime (51%), and aztreonam (27%). Ciprofloxacin (91.5%), imipenem (84.9%) and meropenem (82.1%) were the most effective anti-pseudomonas agents in this study. The results revealed that 88.7% of the isolates were multidrug resistant, 58.25% of those were ESBL positive. Sixteen (26.6%), 9 (15%) and 3 (5%) strains among ESBL-producing strains contained *blaPER-1, blaVEB* and *blaPER-1-blaVEB*, respectively.

**Conclusions:**

This study highlighted the need to establish antimicrobial resistance surveillance networks for *P. aeruginosa* to determine the appropriate empirical treatment regimens. The high prevalence of multidrug resistance and production of ESBLs in *P. aeruginosa* isolates conﬁrms the necessity of protocols considering these issues in the hospitals.

## 1. Background

*Pseudomonas aeruginosa* is an important cause of nosocomial infections, including pneumonia, burn infection, urinary tract infections, meningitis and bacteremia. The infections can be particularly develop to a severe form in immune deficient patients (1). Antibiotics have been used successfully for several decades, but resistance genes have emerged and disseminated particularly in the last few years (2).

Extended-spectrum β-lactamases (ESBLs) mediate resistance to various broad-spectrum cephalosporins, including cefotaxime, ceftriaxone, ceftazidime, and aztreonam (3). These enzymes originally collected from *Klebsiella* *pneumoniae* and *Escherichia coli* and recently from *P*.* aeruginosa *(4-7). Most of the methods for detection of ESBLs are used in bacterial species such as *Klebsiella* and *E. coli* lacking chromosomal β-lactamase activity (8, 9), However, the detection of ESBL production in *P. aeruginosa* has some difficulties, because this bacterium not only has an inducible *AmpC* enzyme but also has an efﬂux-mediated resistance and a higher degree of impermeability than *Enterobacteriaceae *(10, 11). The PER-1 and VEB-1 type ESBLs belong to class A of β- lactamases and is associated with high level of resistance to cephems, monobactams and ceftazidime (12, 13). 

## 2. Objectives

The aim of this study was to determine the antibacterial resistance patterns and prevalence of *PER-1* and VEB type ESBLs among *P. aeruginosa* isolated from patients in west of Iran.

## 3. Materials and Methods

### 3.1. Bacterial Isolates

A total of 106 isolates of *P. aeruginosa* recovered from various clinical specimens (Table 1) in Beasat Teaching Hospital at the Hamadan University of Medical Sciences during a 7-month study in 2009. The identiﬁcation were carried out by colonial morphology, positive oxidase test, pigment formation; growth test at 42ºC on nutrient agar, Gram staining and motility test. 

**Table 1.** Distribution of *P. seudomonas aeruginosa* by Site of Isolation

Source	Isolates, No. (%)
**Burn wounds**	55 (51.9)
**Trashes**	20 (18.9)
**Urine**	12 (11.3)
**Blood**	7 (6.6)
**Feces**	6 (5.7)
**Sputum**	5 (4.7)
**CSF^a^**	1 (0.9)
**Total**	106 (100)

^a^ Abbreviation: CFS, Cerebral spinal fluid

### 3.2. Antibiotic Susceptibility Test

Antibiotic susceptibility of the isolates was determined by standard disk diffusion method (14). The following antibiotics were used: gentamicin (30 µg), aztreonam (30 µg), meropenem (10 µg), imipenem (10 µg), amikacin (30 µg), tobramycin (30 µg), piperacillin (30 µg), ceftazidime (30 µg), ciprofloxacin (5 µg), ofloxacin (5 µg), cefepime (30 µg) and cefotaxime (30 µg) (Himedia, India). *P*.* aeruginosa* ATCC 27853 was used as control. The results were interpreted according to the clinical and laboratory standards institute (CLSI) (15).

### 3.3. Phenotypic Detection of Beta-Lactamase

The isolates were tested for the ESBLs production by using combine disk test (CDT) as CLSI recommendations. CDT were performed on ceftazidime, cefotaxime, cefepime and aztreonam resistant strains by placing disks of ceftazidime, and cefotaxime (30 µg each) at a 20 mm distance from a disk containing ceftazidime-clavulanic acid (30/10μg), cefotaxime-clavulanic acid (30/10µg) and cefepime-clavulanic acid (30/10 µg) (16). ESBL production was inferred when the cephalosporin inhibitory zones were expanded by the clavulanate. 

### 3.4. PCR Ampliﬁcation

PCR ampliﬁcations were done using specific primers for the β-lactamases PER-1, and VEB-1 genes, as described previously (17). PCR was performed for all ESBL-producers which their resistance to cephalosporins and phenotypic was identified by conﬁrmatory tests. DNA was extracted by the boiling method as previously described (17). The DNA ampliﬁcation program consisted of an initial denaturation (94 °C, 5 minutes) followed by 35 cycles of denaturation (94 °C, 60 seconds), annealing (50 °C for PER-1 and 55 °C for VEB-1, 60 seconds), extension (72 °C, 45 seconds) and a single ﬁnal extension for 5 minutes at 72 °C. Reaction mixtures for PCR contained 1.5mM MgCl_2_, 0.5 mM of each primer, 0.2 mM of dNTPs, 1 U of Taq polymerase, 1X PCR buffer and 2 µL of DNA. Primers PER-F (5-AATTTGGGCTTAGGGCAGAA-3') and PER-R (5'-ATGAATGTCATTATAAAAGC-3') were used for blaPER-1; and VEB-F (5'-CGACTTCCATTTCCCGATGC-3') and VEB-R (5'-GGACTCTGCAACAAAT AC GC-3') were used for blaVEB-1 amplification.

## 4. Results

### 4.1. Antimicrobial Susceptibility Test

Table 2 shows the antimicrobial susceptibility pattern of *P. aeruginosa* strains. Ciprofloxacin (91.5%), imipenem.

(84.9%) and meropenem (82.1%) were the most active antimicrobial agents followed by ofloxacin (67.9% susceptibility). Aztreonam, a monobactam, and amikacin showed antibiotic activity against 66% of the strains. Susceptibility to the cephalosporins was reduced to 37.7% for ceftazidime, followed by cefotaxime (9.4%) and Cefepime (0.9%).

**Table 2.** Antimicrobial Susceptibility Pattern of *P*.* aeruginosa* Strains

Antimicrobials	Resistance, No. (%)	Sensitive, No. (%)	Intermediate, No. (%)
**Amikacin**	32 (30.2)	70 (66)	4 (3.8)
**Ceftazidime**	54 (51)	40 (37.7)	12(11.3)
**Aztreonam**	29 (27.4)	70 (66)	7 (6.6)
**‍Cefepime**	103 (97.2)	1 (0.9)	2 (1.9)
**cefotaxime**	53 (50)	10 (9.4)	43 (40.6)
**Ciprofloxacin**	5 (4.7)	97 (91.5)	4 (3.8)
**Gentamicin**	39 (36.8)	55 (51.9)	12 (11.3)
**Imipenem**	8 (7.5)	90 (84.9)	8 (7.5)
**Meropenem**	14 (13.2)	87 (82.1)	5 (4.7)
**Ofloxacin**	31 (29.2)	72 (67.9)	3 (2.8)
**Piperacillin**	99 (93.4)	7 (6.6)	0
**Tobramycin**	39 (36.8)	67 (63.2)	0

**Figure 1.**
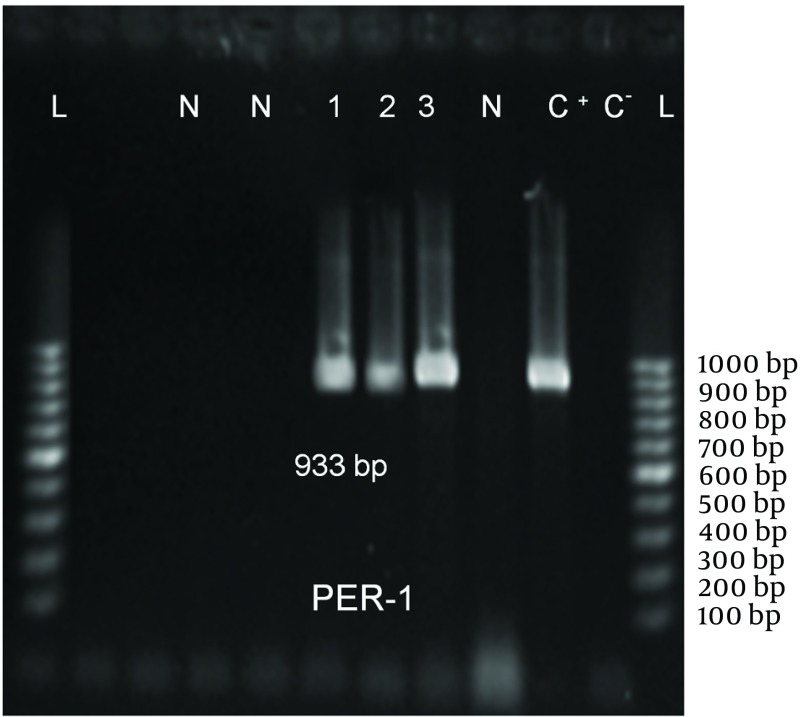
PCR Amplification of *blaPER-1* Gene L: 100 bp DNA ladder, C^-^: negative control, C^+^: positive control, N: PER-1 negative strains, 1–3: PCR products from PER-1positive strains.

**Figure 2.**
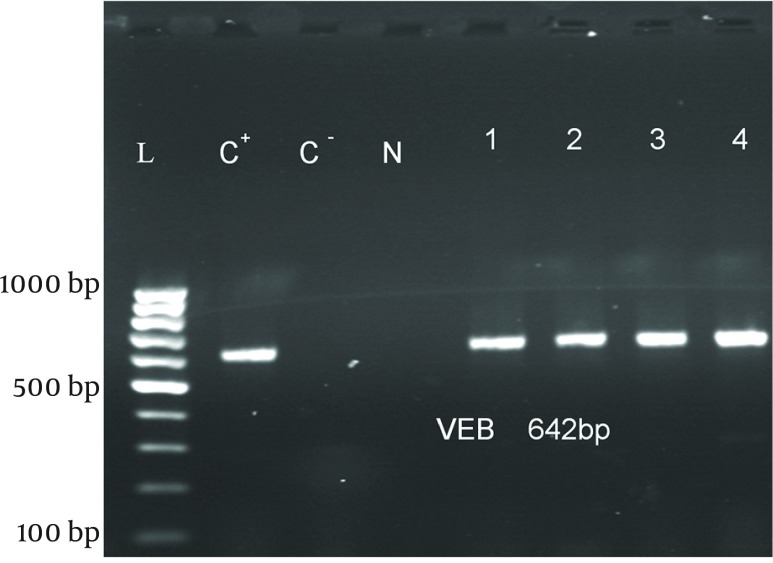
PCR Amplification of *blaVEB-1* Gene L: 100 bp DNA ladder, C^-^: negative control, C^+^: positive control, N: negative strain, 1–4: PCR products fromVEB-1 positive strains.

ESBL Production: Among 106 isolates, 94 (88.7%) were multidrug resistant and 60(58.3%) were putative ESBL producers using phenotypic conﬁrmatory tests (Table 3). Sixteen (26.6%), 9 (15%) and 3 (5%) strains among 60 ESBL-producing strains had *blaPER-1 * (Figure 1), *blaVEB-1* (Figure 2), and *blaPER-1- blaVEB *related genes, respectively (Table 4). 

**Table 3.** Susceptibility Pattern to Antimicrobial Agents in ESBLs and non ESBLs-producing *P*.* aeruginosa* Strains

	ESBL Positive (n=60)			ESBL Negative (n=46)		
Antimicrobials	Resistant, No. (%)	Susceptible, No. (%)	Intermediate, No. (%)	Resistant, No. (%)	Susceptible, No. (%)	Intermediate, No. (%)
**Amikacin**	29 (48.3)	30 (50)	1 (1.7)	4 (9.3)	37 (86)	2 (4.7)
**‍Ceftazidime**	40 (66.7)	18 (30)	2 (3.3)	13 (28.3)	24 (55.2)	6 (13)
**Aztreonam**	25 (41.7)	32 (53.3)	3 (5)	3 (6.97)	39 (90.69)	1 (2.32)
**‍Cefepime**	58 (96.7)	0	2 (3.3)	42 (97.67)	1 (2.32)	0
**cefotaxime**	41 (68.3)	6 (10)	13 (21.7)	12 (27.90)	3 (6.97)	28 (65.11)
**Ciprofloxacin **	5 (8.3)	50 (83.3)	5 (8.3)	0	42 (97.67)	1 (2.32)
**Gentamicin **	32 (53.3)	28 (46.7)	0	7 (16.27)	36 (83.72)	0
**Imipenem**	7 (11.7)	45 (75)	8 (13.3)	1 (2.32)	41 (95.34)	1 (2.32)
**Meropenem**	13 (21.7)	44 (73.3)	3 (5)	2 (4.65)	39 (90.69)	2 (4.65)
**Ofloxacin**	30 (50)	29 (48.3)	1 (1.7)	4 (9.30)	37 (86.04)	2 (4.65)
**Piperacillin**	54 (90)	6 (10)	0	41 (95.34)	2 (4.65)	0
**Tobramycin**	31 (51.6)	28 (46.7)	1 (1.7)	4 (9.30)	39 (90.69)	0

**Table 4.** Association of Antimicrobial Resistance Pattern and ESBL Genotypes

	PER-1:16 (26.6%)			VEB: 9 (15%)		
Antimicrobials	Resistant, No. (%)	Susceptible, No. (%)	Intermediate, No. (%)	Resistant, No. (%)	Susceptible, No. (%)	Intermediate, No. (%)
**Amikacin**	10 (62.5)	5 (31.25)	1(6.25)	5 (55.5)	2 (22.2)	2 (22.2)
**Ceftazidime**	12 (75)	4 (25)	0	9 (100)	0	0
**Aztreonam**	10 (62.5)	4 (25)	2 (12.5)	9 (100)	0	0
**‍Cefepime**	16 (100)	0	0	9 (100)	0	0
**cefotaxime**	87.5 (14)	0	2 (12.5)	9 (100)	0	0
**Ciprofloxacin **	2 (12.5)	13 ( 81.25)	1 (6.25)	2 (22.2)	6 (66.6)	1 (11.11)
**Gentamicin **	10(62.5)	6 ( 37.5 )	0	6 (66.6)	3 (33.3)	0
**Imipenem**	5 (31.25)	5 (31.25)	6 (37.5)	1 (11.11)	6 (66.6)	2 (22.2)
**Meropenem**	7 ( 43.75)	8 (50)	1 (6.25)	6 (66.6)	2 (22.2)	1 (11.11)
**Ofloxacin**	11 (68.8)	5 (31.25)	0	8 (88.8)	1 (11.11)	0
**Piperacillin**	16 (100)	0	0	9 (100)	0	0
**Tobramycin**	10 (62.5)	6 ( 37.5 )	0	6 (66.6)	3 (33.3)	0

## 5. Discussion

*P. aeruginosa* has a high resistance to antibiotics and is a common cause of morbidity and mortality in hospitalized and immunocompromised patients (18). Treatment of *P. aeruginosa* infections is complicated by the inhered and acquired resistance to the most of commonly used antimicrobial agents (19). The results from this study showed the high resistance of *P. aeruginosa* to most of used antimicrobial agents. It was also demonstrated that the prevalence of antibiotic resistance of the isolates was very high in comparison to other studies and most of *P. aeruginosa* isolates (88.7%) were multi-drug resistant (resistant to ≥ 3 different antibiotic classes) (20-25). 

The prevalence of ESBL- producing *P. aeruginosa* isolates in this study was also higher than other investigations (5, 20, 26, 27). Among 60 ESBL-positive strains, 16 (26.6%), 9 (15%) and 3 (5%) contained *PER-1*,* VEB-1* and *PER-1-VEB-1 *genes, respectively. These results indicated that the prevalence of *VEB-1* gene in our area, is higher than Turkey and Korea, but the prevalence of *VEB-1* and *PER-1* genes, is lower than in Thailand (94.44% *blaVEB-1*) and Italy (34.61% *blaPER-1*) (5, 13, 26). Data on the prevalence of ESBL- producing *P. aeruginosa* strains in our area is limited. In the study performed by Shahcheraghi and colleges on *P. aeruginosa* isolates in Tehran, the rate of *blaVEB* and *blaPER* ESBLs were reported 24% and 17%, respectively, that was similar to our results (28). The high prevalence of *PER-1* and *VEB-1* indicated the high resistance to penicillins, ceftazidime and cefotaxime, as reported by other studies (26, 28).

This is the first report about the presence of these enzymes in *P. aeruginosa* isolates from west of Iran. It has shown that ESBL production in strains of *P. aeruginosa* can greatly complicate the clinical management of infection if advanced care is not taken. However, further studies are needed to determine other ESBL- types in *P.aeruginosa* strains in this area and their role in resistance to other antibiotic classes. The results of this study emphasizes on the need for a surveillance network to monitor the trends and emerge of new resistance mechanism in *P. aeruginosa *from different geographic regions. Therefore, the improvement in antibiotic prescription policies and infection control programs are of high necessity to prevent the spread of such resistant infectious agents.
